# Risk factors for the mortality of hepatitis B virus-associated acute-on-chronic liver failure: a systematic review and meta-analysis

**DOI:** 10.1186/s12876-023-02980-4

**Published:** 2023-10-03

**Authors:** Hanyun Tu, Rong Liu, Anni Zhang, Sufei Yang, Chengjiang Liu

**Affiliations:** 1 School of Medicine, Jinan University, Guangzhou, 510632 China; 2 Sichuan Institute of Product Quality Supervision and Inspection, Chengdu, 610100 China; 3 Department of Cardiology, Daping Hospital, Army Medical University), Third Military Medical University, Chongqing, 400042 China; 4 Department of General Medicine, Affiliated Anqing First People’s Hospital of Anhui Medical University, Anqing, 246004 China

**Keywords:** Hepatitis B virus, Acute-on-chronic liver failure, Mortality, Risk factors, Systematic review, Meta-analysis

## Abstract

**Background:**

Hepatitis B virus-associated acute-on-chronic liver failure (HBV-ACLF) has been confirmed as a prevalent form of end-stage liver disease in people subjected to chronic HBV infection. However, there has been rare in-depth research on the risk factors for the mortality of HBV-ACLF. This study aimed at determining the risk factors for the mortality of HBV-ACLF.

**Methods:**

The relevant research was selected from four electronic databases that have been published as of August 2023. The existing research was reviewed in accordance with the inclusion and exclusion criteria. The level of quality of previous research was evaluated using the Newcastle–Ottawa scale. Moreover, a pooled estimate of the odds ratios (ORs) with their associated 95% confidence intervals (CIs) was provided through a meta-analysis. The data were combined, and the risk variables that at least two studies had considered were analyzed. The publication bias was examined through Egger's test and Begg's test.

**Results:**

Twenty two studies that conformed to the inclusion criteria were selected from 560 trials. Eight risk variables in terms of HBV-ACLF mortality were determined, which covered INR (OR = 1.923, 95% CI = 1.664–2.221, *P* < 0.001), Monocytes (OR = 1.201, 95% CI = 1.113–1.296, *P* < 0.001), Cirrhosis (OR = 1.432, 95% CI = 1.210–1.696, *P *< 0.001), HE (OR = 2.553, 95% CI = 1.968–3.312, *P* < 0.001), HE grade (OR = 2.059, 95% CI = 1.561–2.717, *P* < 0.001), SBP (OR = 1.383, 95% CI = 1.080–1.769, *P* = 0.010), Hyponatremia (OR = 1.941, 95% CI = 1.614–2.334, *P* < 0.001), as well as HRS (OR = 2.610, 95% CI = 1.669–4.080, *P* < 0.001).

**Conclusion:**

The most significant risk factors for HBV-ACLF mortality comprise HRS, HE, and HE grade, followed by INR and hyponatremia. The Monocytes, cirrhosis, and SBP have been confirmed as the additional key risk factors for HBV-ACLF mortality.

**Supplementary Information:**

The online version contains supplementary material available at 10.1186/s12876-023-02980-4.

## Introduction

Despite the availability of effective vaccinations against Hepatitis B virus (HBV) infection, this infection continues to be a serious public health problem worldwide [1]. 257 million people worldwide are subjected to chronic HBV infection, and 60,000 people worldwide die from chronic hepatitis B each year, as estimated by The World Health Organization (WHO). China has been reported as one of the areas with a high incidence of HBV, since nearly 30 million people in China are suffering from chronic HBV infection [2]. Approximately 20% of HBV chronic infection patients will progress to acute-on-chronic liver failure (ACLF), [3, 4] which is a devastating entity [5]. The core of ACLF suggests that a patient subjected to chronic liver disease will abruptly lose their present level of liver function in the event of an acute insult [6]. It is noteworthy that ACLF may occur at any point during chronic hepatitis B development [7].

To be more specific, hepatitis B virus-associated acute-on-chronic liver failure (HBV-ACLF) has been confirmed as a prevalent pattern of end-stage liver disease in patients subjected to chronic HBV infection, which is manifested as a fast worsening of preexisting chronic liver problems with multisystem organ failure [4, 8]. HBV-ACLF exhibits substantial short-term mortality, ranging from 40 to 80%, in accordance with the diagnostic criteria [9–11]. It has been widely recognized that scores of variables can facilitate the advancement of HBV-ACLF. Numerous variables (e.g., infection [12, 13], COSSH-ACLF grade [14], and gastrointestinal bleeding [15]) have been reported as the risk factors for the mortality of HBV-ACLF in individual retrospective research. On that basis, discovering HBV-ACLF and encouraging the early diagnosis and treatment of this failure take on critical significance to lowering the mortality of HBV-ACLF.

Nevertheless, the effect and compelling evidence of possible risk variables have not been validated thus far. Moreover, the collection and analysis of the risk variables for HBV-ACLF mortality have never been the subject of a meta-analysis. Inspired by the above-described analysis, we conducted a systematic review and meta-analysis to determine and evaluate the risk factors for the mortality of HBV-ACLF.

## Methods

### Protocol

A systematic review was conducted using a specified protocol according to the Preferred Reporting Items for Systematic Reviews and Meta-Analyses (PRISMA) statement [16]. Our review has been duly registered with the PROSPERO database under the registration number CRD42023413223 [17]. As shown in Supplementary File 1, we present this article in accordance with the PRISMA reporting checklist.

### Data source collection

The electronic databases of PubMed, the Cochrane Library, Embase, and Web of Science were searched from inception till August 2023. Table 1 lists the search terms on PubMed. The search results were imported into Endnote 20.4 (USA, 2020, Thomson Corp) for management.

**Table 1:** Keywords used in The PubMed database

Search terms	
#1	Acute-On-Chronic Liver Failure [Mesh Terms]
#2	ACLF [All Fields]
#3	#1 OR #2
#4	Hepatitis B virus [Mesh Terms]
#5	HBV [All Fields]
#6	#4 OR #5
#7	#3 AND #6
#8	HBV-ACLF [All Fields]
#9	hepatitis B virus-associated acute-on-chronic liver failure [All Fields]
#10	hepatitis B virus-related acute-on-chronic liver failure [All Fields]
#11	#7 OR #8 OR #9 OR #10
#12	Risk Factors [Mesh Terms]
#13	risk factor [All Fields]
#14	factor, risk [All Fields]
#15	factors, risk [All Fields]
#16	#12 OR #13 OR #14 OR #15
#17	Mortality [Mesh Terms]
#18	death rate [All Fields]
#19	#17 OR #18
#20	#11 AND #16 AND #19

### Inclusion and exclusion criteria

The inclusion criteria are presented as follows: (1) The design of the research was a case–control or cohort study. (2) The research had something to do with the mortality risk factors in HBV-ACLF patients. (3) The Asian Pacific Association's or Chinese Medical Association's or European Association’s suggested criteria for the research of the liver were used to define HBV-ACLF [18–22]. (4) The risk factors for the mortality of HBV-ACLF were obtained through this study.

The exclusion criteria are elucidated as follows: (1) Duplicated studies, (2) Reviews, meta-analyses, suggestions, animal experiments, or reports of conference, (3) No diagnostic criteria for HBV-ACLF, (4) Not enough information was available to calculate odds ratios (OR) and 95% confidence intervals (CI) for the mortality of HBV-ACLF.

### Data extraction

Three reviewers (H.T., R.L. and S.Y.) extracted the fundamental materials from the selected articles. The extraction findings were assessed by three reviewers (H.T., R.L. and A.Z.), and any disputes were settled through conversation. The extracted data included author, year of publication, research duration, province, study design, sample size, age and sex as well as the data for the risk factors.

### Quality assessment

Two reviewers (H.T., S.Y.) independently evaluated the quality of these studies. As depicted in Table 2, the Newcastle–Ottawa Scale (NOS) ranges from zero to a maximum achievable score of nine; it elucidates participant selection, comparability between groups, and exposure or result evaluation. The quality evaluation was performed using the NOS. The included studies were all considered with high quality (6–9 points) [23].

**Table 2:** The Newcastle–Ottawa quality assessment scale for included studies

Included studies	Participant selection	Comparability between groups	Exposure or result evaluation	Total points
Zhang, X., et al. 2022 [24]	3	2	3	8
Yang, J., et al. 2022 [25]	3	2	3	8
Weng, W.Z., et al. 2022 [15]	3	2	2	7
Wang, L., et al. 2022 [26]	3	2	2	7
Lin, L., et al. 2022 [27]	3	2	3	8
Xue, R., et al. 2021 [28]	3	1	3	7
Xiao, L.L., et al. 2021 [14]	3	1	2	6
Xiao, L., et al. 2021 [29]	3	2	3	8
Sun, J., et al. 2021 [30]	3	2	3	8
Hu, H., et al. 2021 [31]	3	2	3	8
Zhai, X.R., et al. 2020 [13]	3	2	2	7
Jia, L., et al. 2020 [12]	3	2	2	7
Yi, Z.Q., et al. 2015 [32]	3	1	2	6
Qin, G., et al. 2014 [33]	3	1	3	7
Hou, Y., et al. 2020 [34]	3	2	2	7
Lu, J., et al. 2019 [35]	3	1	2	6
Li, T.P., et al. 2019 [36]	3	2	3	8
Shi, X., et al. 2016 [37]	3	1	2	6
Zhang, G.L., et al. 2016 [38]	3	1	2	6
Gao, F., et al. 2017 [39]	3	2	3	8
Liu, L., et al. 2018 [40]	3	1	3	7
Li, X., et al. 2017 [41]	3	2	2	7

### Synthesis and analysis of data

The results included in the included studies were classified using the narrative synthesis method, and the structure was designed in accordance with the characteristics of the subjects and the distribution of potential risk factors and results. A pooled estimate of the ORs with their associated 95% CIs was provided through a meta-analysis, in which the data from at least two studies could be integrated. Hazard ratio (HR) and OR were considered to be equal when less than 10. As a result, HR and OR were incorporated into OR.

The relative probability of potential risk factors for the mortality of HBV-ACLF patients was determined using STATA 15.1 (USA, 2017, Stata Corp). A fixed-effects model was employed under I^2^ > 50% and *P* < 0.05. In contrast, the 95% CI of the homogenous data was accounted for using a random effects model. The factor was considered the risk factor of mortality in HBV-ACLF if the combined OR of an associated factor exceeded 1.200, and the lower limit of 95% CI reached over 1.00. Heterogeneity between studies was evaluated in accordance with the I^2^ statistic and based on the *P* value of the chi-squared test. Begg’s test and Egger’s test were performed to evaluate the publication bias. *p* < 0.05 indicated that a potential publication bias was present.

## Results

### Study selection

**Fig. 1:**
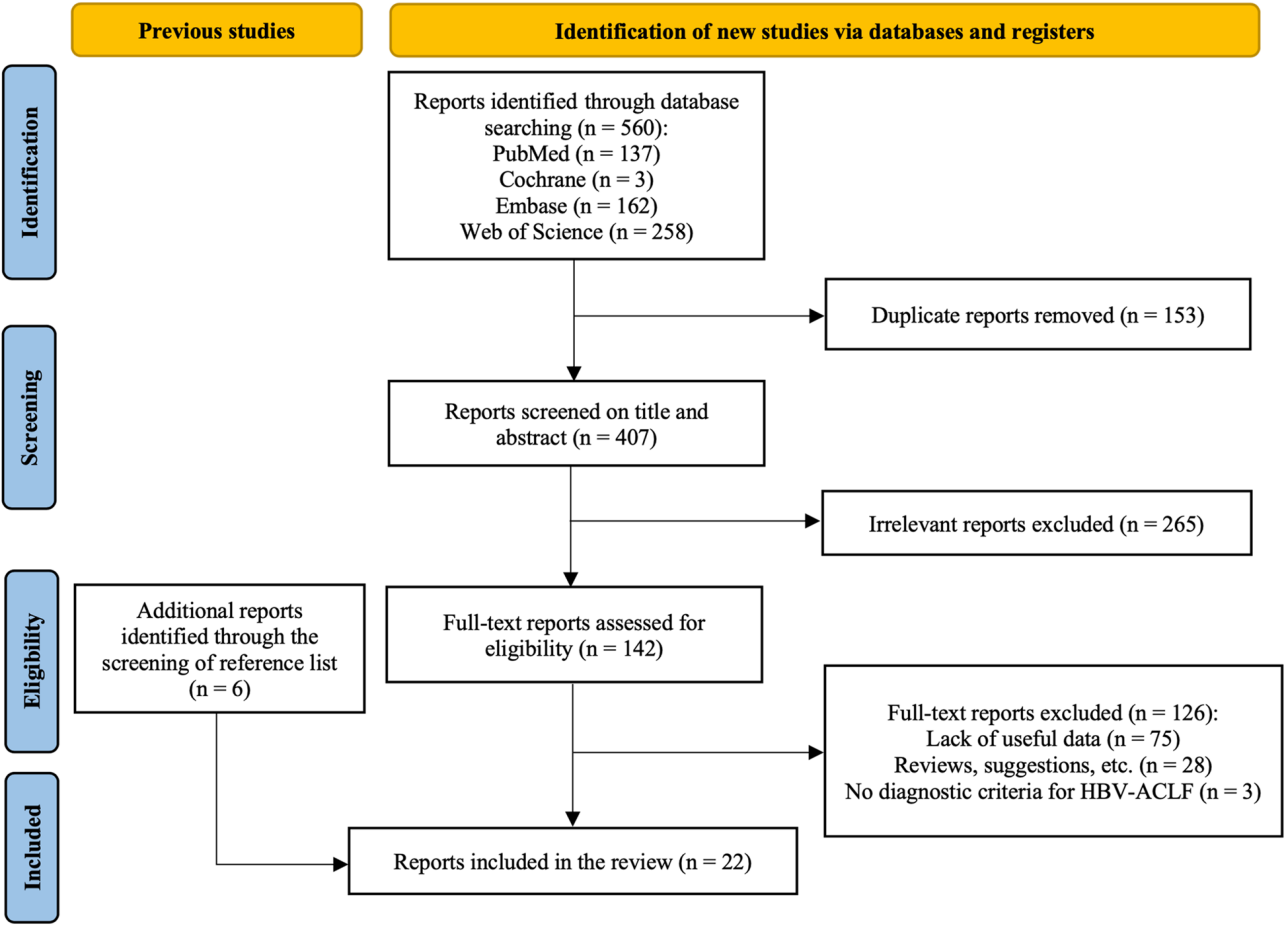
Preferred reporting items for systematic reviews and meta-analyses (PRISMA) flow diagram of the search strategy

Figure 1 presents the method for selecting the studies, and the outcomes of the literature search are shown. 560 items in all were located via electronic database searching. After browsing the titles and abstracts of 407 studies after duplication removal, 142 potentially eligible studies remained for further investigation. By carefully examining the reference lists from the retrieved publications, we also discovered six more studies. 22 studies that conformed to the predetermined criteria were added after thorough evaluations.

### Study characteristics

Table 3 lists the features of the studies that were employed as the risk factors for the mortality of HBV-ACLF. 5 studies were case–control studies [12, 27, 33, 35, 36], and the others were cohort studies [13–15, 24–26, 28–32, 34, 37–41]. In general, 10,878 cases of HBV-ACLF were covered in the cohort studies, which were published between 2015 and 2022. A total of 681 cases were included in the case–control studies that were published between 2014 and 2022.

**Table 3:** Characteristics of included studies

Authors, year of publication, Country	Study design, period	Newcastle–Ottawa score	Sample size	Age(years)	Sex	Risk factors found for the mortality of HBV-ACLF	Measures of associations	Selection criteria for HBV-ACLF
Zhang, X., et al. 2022 China [24]	Cohort 2013–2021	8 points	*n* = 163	46 (37–56)	Male = 149	-	HR	APASL
					Female = 14			
Yang, J., et al. 2022 China [25]	Cohort 2019–2020	8 points	*n* = 180	NA	Male = 19	Monocytes	OR	APASL
					Female = 161			
Weng, W.Z., et al. 2022 China [15]	Cohort 2010–2018	7 points	*n* = 2166	45 (37–54)	Male = 1914	Cirrhosis, SBP, HE, HRS	HR	CMASL
					Female = 252			
Wang, L., et al. 2022 China [26]	Cohort 2014–2018	7 points	*n* = 1177	45.06 (10.57)	Male = 124	Cirrhosis, HE	OR	APASL
					Female = 1153			
Lin, L., et al. 2022 China [27]	Case control 2015–2021	8 points	*n* = 116	NA	Male = 98	HE, SBP, INR	OR	APASL
					Female = 18			
Xue, R., et al. 2021 China [28]	Cohort 2014–2018	7 points	*n* = 171	45.17 (12.49)	Male = 151	HE, Monocytes	OR	APASL
					Female = 20			
Xiao, L.L., et al. 2021 China [14]	Cohort 2008–2015	6 points	*n* = 1973	NA	Male = 1641	INR	OR	APASL
					Female = 332			
Xiao, L., et al. 2021 China [29]	Cohort 2018–2020	8 points	*n *= 175	NA	Male = 146	INR, HE grade	OR	APASL
					Female = 29			
Sun, J., et al. 2021 China [30]	Cohort 2013–2019	8 points	*n *= 494	45 (37–55)	Male = 421	HE, INR, HE grade	HR	APASL
					Female = 73			
Hu, H., et al. 2021 China [31]	Cohort 2017–2020	8 points	n = 96	NA	Male = 79	INR	OR	CMASL
					Female = 17			
Zhai, X.R., et al. 2020 China [13]	Cohort 2015–2017	7 points	*n* = 289	NA	Male = 236	INR, HE	HR	EASL
					Female = 53			
Jia, L., et al. 2020 China [12]	Case control 2013–2015	7 points	*n* = 171	45.1 (12.3)	Male = 152	Cirrhosis, Monocytes, INR, HE, HRS	HR	APASL
					Female = 19			
Yi, Z.Q., et al. 2015 China [32]	Cohort 2008–2011	6 points	*n* = 392	NA	Male = 323	HE, INR	OR	APASL
					Female = 69			
Qin, G., et al. 2014 China [33]	Case control 2003–2007	7 points	*n *= 234	NA	Male = 180	INR, Cirrhosis, HE, HRS, SBP	HR	APASL
					Female = 54			
Hou, Y., et al. 2020 China [34]	Cohort 2008–2016	7 points	*n* = 684	43.9 (11.6)	Male = 582	Hyponatremia, SBP, HE, HRS, INR	OR	APASL
					Female = 102			
Lu, J., et al. 2019 China [35]	Case control 2015–2017	6 points	*n* = 54	46.72 (12.26)	Male = 48	HRS, HE, SBP	OR	APASL
					Female = 6			
Li, T.P., et al. 2019 China [36]	Case control 2017–2018	8 points	*n* = 106	NA	Male = 87	-	OR	APASL
					Female = 19			
Shi, X., et al. 2016 China [37]	Cohort 2010–2015	6 points	*n* = 1167	NA	Male = 996	HE	OR	CMASL
					Female = 171			
Zhang, G.L., et al. 2016 China [38]	Cohort 2009–2010	6 points	*n* = 65	41.52 (1.47)	Male = 62	-	HR	APASL
					Female = 3			
Gao, F., et al. 2017 China [39]	Cohort 2003–2013	8 points	*n* = 573	43.5 (11.5)	Male = 478	Hyponatremia, SBP, HE, HRS, INR	HR	APASL
					Female = 95			
Liu, L., et al. 2018 China [40]	Cohort 2009–2016	7 points	*n* = 355	NA	Male = 290	SBP	OR	APASL
					Female = 65			
Li, X., et al. 2017 China [41]	Cohort 2000–2015	7 points	*n* = 758	NA	Male = 643	INR	HR	APASL
					Female = 115			

### Risk factors of mortality in HBV-ACLF patients

**Fig. 2:**
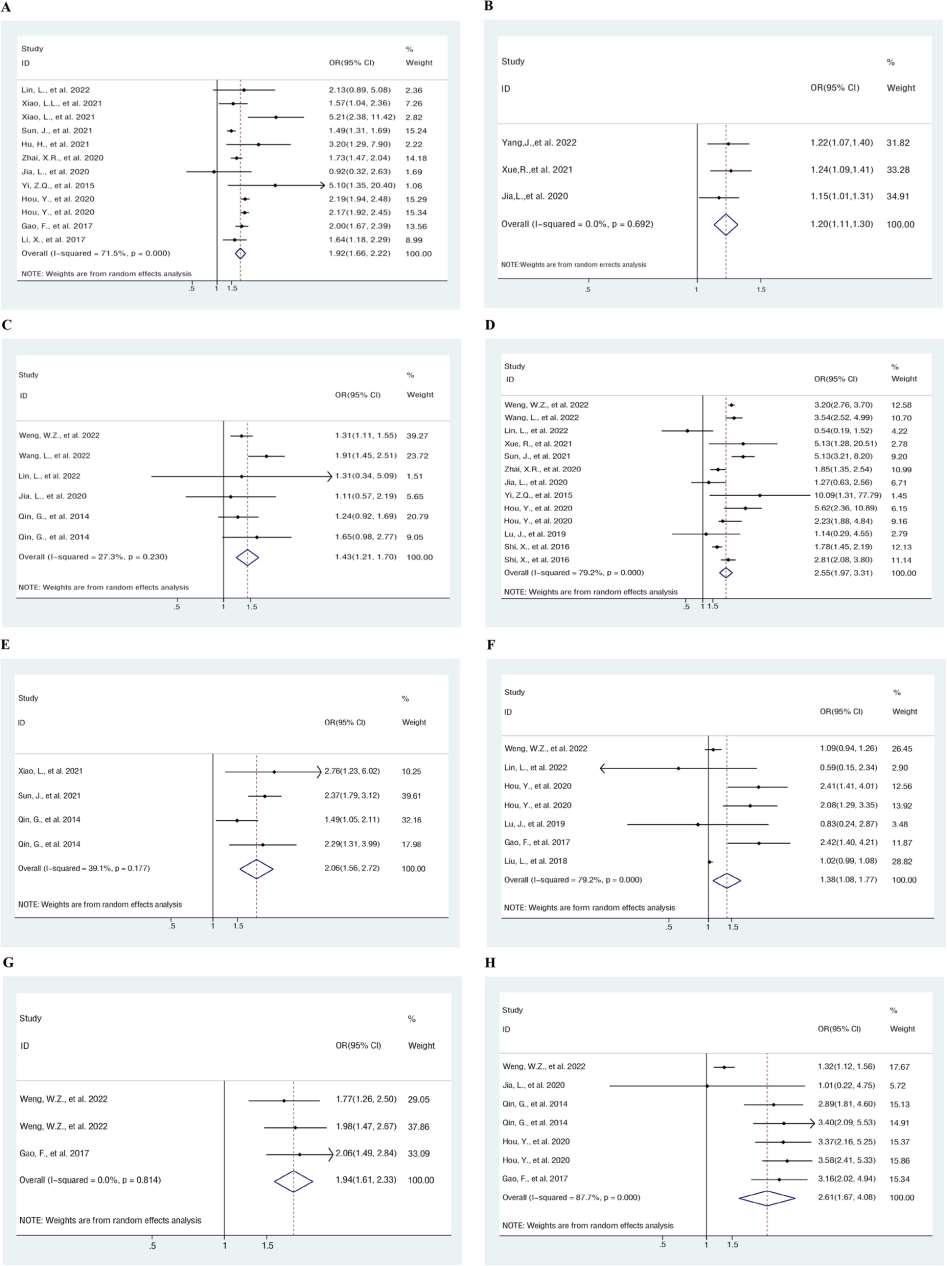
Forest plot synthesizing the overall odds ratio for risk factors for the mortality of HBV-ACLF. **A** Forest plot of INR. **B** Forest plot of Monocytes. **C** Forest plot of cirrhosis. **D** Forest plot of HE. **E** Forest plot of HE grade. **F** Forest plot of SBP. **G** Forest plot of hyponatremia. **H** Forest plot of HRS

The mortality risk variables for HBV-ACLF patients are listed in Table 4 of the meta-analysis. Among the risk factors, the international normalized ratio (INR), monocytes and hepatic encephalopathy grade (HE grade) were continuous variables, and cirrhosis, HE, spontaneous bacterial peritonitis (SBP), hyponatremia, and hepatorenal syndrome (HRS) were binary. A total of 8 risk factors (i.e., INR, Monocytes, Cirrhosis, HE, HE grade, SBP, Hyponatremia and HRS) for HBV-ACLF mortality exerted a substantial impact. Figure 2A-H successively presents the forest plot of INR, monocytes, cirrhosis, HE, HE grade, SBP, hyponatremia and HRS that illustrates how the risk factors for HBV-ACLF are correlated with death.

**Table 4:** Meta-analysis for risk factors of mortality in HBV-ACLF

Risk factors	Combination studies	Heterogeneity of study design		Analysis model	Results of meta-analysis		Begg’s test	Egger’s test
		P	I^2^		OR (95%CI)	P		
INR	11	< 0.001	71.5%	Random	1.923 (1.664, 2.221)	< 0.001	1.000	0.628
Monocytes	3	0.692	0.0%	Fixed	1.201 (1.113, 1.296)	< 0.001	1.000	0.449
Cirrhosis	5	0.230	27.3%	Fixed	1.432 (1.210, 1.696)	< 0.001	0.707	0.882
HE	11	< 0.001	79.2%	Random	2.553 (1.968, 3.312)	< 0.001	1.000	0.801
HE grade	3	0.177	39.1%	Fixed	2.059 (1.561, 2.717)	< 0.001	1.000	0.817
SBP	6	< 0.001	79.2%	Random	1.383 (1.080, 1.769)	0.010	1.000	0.115
Hyponatremia	2	0.814	0.0%	Fixed	1.941 (1.614, 2.334)	< 0.001	1.000	0.521
HRS	5	< 0.001	87.7%	Random	2.610 (1.669, 4.080)	< 0.001	0.548	0.079

### Laboratory indicators

The laboratory indicators in the study comprised INR and Monocytes. As indicated by the combination and result analysis of 11 selected studies, INR was a crucial risk factor for HBV-ACLF death (OR = 1.923, 95% CI = 1.664–2.221, *P* < 0.001). Three studies have reported that Monocytes is a risk factor for the mortality of HBV-ACLF (OR = 1.201, 95% CI = 1.113–1.296, *P* < 0.001).

### Complication factors

Cirrhosis, hyponatremia, HE, SBP, and HRS were among the consequences in this study. The findings of a meta-analysis of five studies suggested that cirrhosis increased the probability of dying from HBV-ACLF (OR = 1.432, 95% CI = 1.210–1.696, *P* < 0.001). In 11 studies, HE was reported as the primary risk factor for HBV-ACLF mortality (OR = 2.553, 95% CI = 1.968–3.312, *P* < 0.001); in three studies, the risk of death was elevated with the rise of the HE grade (OR = 2.059, 95% CI = 1.561–2.717, *P* < 0.001). Six studies indicated SBP as risk factors for HBV-ACLF mortality, respectively (OR = 1.383, 95% CI = 1.080–1.769, *P* = 0.010). In two trials, hyponatremia was reported as a meaningful risk factor for HBV-ACLF mortality (OR = 1.941, 95% CI = 1.614–2.334, *P* < 0.001). Given the finding of the meta-analysis, HRS was confirmed as the most critical risk factor for HBV-ACLF mortality (OR = 2.610, 95% CI = 1.669–4.080, *P* < 0.001).

## Publication bias

Begg’s Test and Egger’s Test were performed to assess the publication bias of the included studies. Table 4 presents the results. In line with the findings of this meta-analysis, none of the risk variables had any publication bias (*P* > 0.05).

## Discussion

A substantial short-term mortality rate is present in the acute phase of HBV-ACLF. In this systematic review and meta-analysis, 22 case–control or cohort studies published from 2014 to 2022 were included and analyzed, with a sample size of 11,559 cases. As indicated by the results, INR, Monocytes, cirrhosis, HE, HE grade, SBP, hyponatremia, and HRS may serve as risk factors for the mortality of HBV-ACLF patients, such that evidence can be provided for reducing mortality.

This study has suggested that INR is the most relevant risk factor for the mortality of HBV-ACLF among the laboratory indicators. HBV-ACLF refers to a type of severe liver dysfunction. When liver functions are significantly compromised, plasminogen, a protein unique to the liver, would dramatically decline along with other coagulation factors. In theory, a drop in the plasminogen levels in individuals with HBV-ACLF should be accompanied by an elevation of the INR value due to the liver's decreased ability to synthesize proteins. HBV-ACLF patients will have greater mortality since the danger of excessive bleeding increases with the INR values [42]. Thus, it may be necessary that proper measures should be adopted to get the INR value back to normal, so as to lessen the effect of INR on the mortality of HBV-ACLF.

Moreover, systemic inflammatory response syndrome (SIRS) was reported as the main contributor by speculating on the potential mechanism of Monocytes as a risk factor for HBV-ACLF mortality. ACLF is considered a syndrome of innate immune dysfunction. As revealed by the pathophysiological basis of ACLF, significant tissue and organ damage primarily arises from a systemic hyperinflammatory condition. Tissue damage is made worse by the significant production of inflammatory mediators that causes immune-mediated tissue damage [43]. Dysfunction of monocytes and macrophages significantly affects the development of ACLF illness. Innate effector cells are drawn to the wounded liver since Kupffer cells are activated by pathogen and damage-associated molecular patterns. Early monocyte infiltration may facilitate local tissue degradation during the propagation phase and produce pro-inflammatory cytokines that trigger SIRS. After local reprogramming is completed, monocytes recruited in the hepatic milieu develop into macrophages to support resolution reactions and preserve tissue integrity [44, 45]. On that basis, Monocytes serves as a vital risk factor for the mortality of HBV-ACLF.

As indicated by the analysis of the mechanism for complication risk factors, cirrhosis arising from by HBV and renal impairment can account for the three complication risk factors listed. HBV has been reported as the main cause of liver cirrhosis among the Chinese [46]. In general, functional renal impairment occurs in severe cirrhosis patients [47]. Existing research has suggested that HBV-ACLF patients with cirrhosis are subjected to more extrahepatic organ failures and a less favorable outcome, i.e., more mortality [48]. Renal failure, the most frequent extrahepatic organ failure, is associated with systemic circulatory insufficiency and reduced renal perfusion in liver cirrhosis patients. Nephritis is likely to jeopardize renal microcirculation and cell function. Thus, it may also directly affect the development of the condition [49]. Cirrhosis patients often develop ascites. For patients with ascites attributed to cirrhosis, SBP has been reported as one of the most prominent infections, and it leads to dangerous consequences. SBP refers to an infection that develops in the abdominal cavity without a direct bacterial cause in nearby abdominal organs [50, 51]. The mortality may be increased under a higher frequency of detection of irreversible renal impairment, HRS, and gram-positive bacteria in ACLF patients with SBP [52, 53]. Previous research has suggested that ACLF patients with SBP have a 50% chance of dying within 28 days [54, 55]. Hyponatremia increased renal sodium retention and solute-free water retention can cause ascites and hyponatremia, and they are considered the characteristics of renal failure in ACLF patients. As revealed by an early investigation, the existence or lack of concurrent hyponatremia deeply affects the prognosis of ACLF patients. ACLF and hyponatremia patients exhibited a lower three-month death expectancy than those patients subjected to ACLF and hyponatremia [47, 56]. Hyponatremia in cirrhotic patients has long been considered a distinct risk factor for death. Hyponatremia has long been recognized as a separate risk factor for the mortality of cirrhotic patients. A secondary hyperaldosteronism condition has been reported in cirrhosis patients. Aldosterone, a mineralocorticoid hormone, can increase the number of open sodium channels to facilitate sodium reabsorption and potassium secretion. Furthermore, patients with advanced cirrhosis have a gradual vasodilatory condition that reduces the effective arterial blood volume and activation of the renin–angiotensin–aldosterone system (RAAS). Antidiuretic hormone and aldosterone result in ascites and hyponatremia through the retaining process of water and sodium [57].

Under the effect of renal failure, HRS, as an underlying mechanism, becomes a risk factor more pertinent to the mortality of HBV-ACLF compared with cirrhosis and renal impairment. The kidney is the organ in which extrahepatic organ failure most often occurs [58, 59]. HRS refers to a type of functional renal failure characterized by inadequate central filling, which arises from inadequate cardiac output, dilated visceral arteries, and systemic peripheral blood vessels. Arterial dilatation has been confirmed as the main pathogenic event in HRS, reducing effective blood volume, steadily activating the RAAS, and resulting in renal vasoconstriction by activating the sympathetic nervous system [60–62]. HRS refers to a severe HBV-ACLF complication and serves as a strong predictor of high short-term and long-term death. Existing research has suggested that the 30-day mortality rate of HBV-ACLF patients without HRS just reaches 13.95%; besides, for HBV-ACLF patients with HRS, 57.14% pass away during the hospital's 30-day observation period [62]. Accordingly, it serves as a reminder that effective surveillance plans and prompt therapies for aberrant renal behavior are crucial.

HE serves as the second critical risk factor for the mortality of HBV-ACLF, and it is non-negligible. The presence of hepatic encephalopathy (HE) and the progression of its grade can lead to the increased death rate for ACLF patients [63]. In ACLF, HE has a significant mortality rate that is not reliant on other organ failures. Existing studies have suggested that ACLF patients have obviously increased intracranial pressure and mortality from cerebral edema [64]. Since glutamine synthetase is active in ACLF, hyperammonemia can increase intracellular glutamine content and osmotic pressure concentration, resulting in astrocyte swelling and increased oxidative stress. Hyponatremia is likely to cause a second osmotic assault on astrocytes, exacerbating intracellular edema [47]. A tiny percentage of ACLF patients develop symptoms (e.g., brain swelling). Younger cirrhotic, accompanied by severe liver dysfunction, a widespread inflammatory response, bacterial infections, excessive drinking, and dilutional hyponatremia are more susceptible to HE linked with ACLF [65]. Even though the exact mechanism of HE in ACLF remains unclear, ammonia and the body's inflammatory response may serve as key players [66].

This study still had several limitations. First, four databases were only searched, such that we could have omitted some relevant studies and data. Second, some included studies had a small size of sample that tended to generate bias and affected the reliability of the conclusion. Third, the results may deviate due to the intricate relationships between different risk variables and other factors.

## Conclusions

In brief, HRS, HE, and HE grade are the most relevant risk factors for the mortality of HBV-ACLF, followed by INR and hyponatremia. Other important risk factors for the mortality of HBV-ACLF comprise Monocytes, cirrhosis, and SBP. Attention should be placed on all risk factors, and a wide variety of preventative or treatment methods should be implemented for distinct risk variables to lower the mortality of HBV-ACLF.

### Supplementary Information

**Additional file 1. **PRISMA Checklist.

## Data Availability

All data generated or analyzed during this study are included in this published article and its supplementary information files.

## Abbreviations

HBV-ACLF: Hepatitis B virus-associated acute-on-chronic liver failure
Cis: Confidence intervals
OR: Odds ratio
HR: Hazard ratio
HBV: Hepatitis B virus
ACLF: Acute-on-chronic liver failure
WHO: The World Health Organization
INR: The international normalized ratio
HE: Hepatic encephalopathy
SBP: Spontaneous bacterial peritonitis
HRS: Hepatorenal syndrome
APASL: The Asian Pacific association for the study of the liver
CMASL: The Chinese Medical association for the study of the liver
EASL: The European association for the study of the liver
SIRS: Systemic inflammatory response syndrome
RAAS: The renin–angiotensin–aldosterone system
